# 

**Published:** 1888-09-08

**Authors:** 


### CONTENTS.

**Children and Crime** - - - - - - - 365
**The Art of Embalming** - - - - - - - 366
**The Claims of Hospitals.** By R. BRUDENELL CARTER, F.R.C.S. - - - - - - - 367
**The Doctor's Pulpit.**—Marriage and Morals - - - 369
**Hints for the Sick-room.** By M. M. BASIL, M.A., M.B. 370
**In and Out Among the Hospitals.** By a SECRETARY of TEN YEARS' STANDING - - - - - - 371
**Notes and News** - - - - - - - 372
**Everybody's Page** - - - - - - - 374
**Annotations.**—The National Pension Fund and Its One Adverse Critic.—The Stable Door.—The Cancer Hospital at Brompton 375
**The King of Causes of Disease.** By Sir WILLIAM MOORE, K.C.I.E. - - - - - - - 376
**An Ishmaelite.** By LESLIE KEITH, Author of "The Chilcotes," "East and West," etc. - - - - - - - 377
**Within the Wards.**—Tuberculosis.—Squinting and Worms.—Saccharin for Diabetics.—Galvanic Treatment of Parasitic Diseases of the Skin - - - - - - - 379
**Amusements with Profit for all Ages** - - - 380

### EXTRA SUPPLEMENT.—THE NURSING MIRROR.

En Passant.—Hol'day House.—Personal Items.—Knowledge for Nurses.—The New York Climate.—A Suggestion for Sisters.—Nurses on the Continent.—The Police Courts Again - lxxxix
Original Articles.—Sick Nursing Among the Poor—An Amateur's View - - - - - - - - xc
Letters from a Probationer.—II. - - - - - - xci
Notes from Australia - - - - - - - xci
Everybody's Opinion.—The Zenana Mission.—The Question of Courage - - - - - - - - xcii
Appointments—Vacancies—Notes and Oueries - - - xcii

